# Crystallization and crystallographic studies of kallistatin

**DOI:** 10.1107/S2053230X15012893

**Published:** 2015-08-25

**Authors:** Fang Lin, Aiwu Zhou, Zhenquan Wei

**Affiliations:** a Hongqiao International Institute of Medicine, Shanghai Tongren Hospital and Faculty of Basic Medicine, and Department of Pathophysiology, Shanghai Jiaotong University School of Medicine, (Room 1006, Building 2, No 280, South Chongqing Road), Shanghai 200025, People’s Republic of China

**Keywords:** kallistatin, tissue kallikrein, crystallization, serpins, heparin

## Abstract

Kallistatin is a serine protease inhibitor (serpin) which specifically inhibits human tissue kallikrein; however, its inhibitory activity is inhibited by heparin. In order to elucidate the underlying mechanism, recombinant human kallistatin was prepared in *Escherichia coli* and the protein was crystallized by the sitting-drop vapour-diffusion method. X-ray diffraction data were collected to 1.9 Å resolution. The crystals were found to belong to space group *P*6_1_, with unit-cell parameters *a* = 113.51, *b* = 113.51, *c* = 76.17 Å. Initial analysis indicated that the crystallized kallistatin was in a relaxed conformation, with its reactive-centre loop inserted in the central β-sheet.

## Abstract 2

The crystallization of human kallistatin in the relaxed conformation is reported.

## 1. Introduction   

Kallistatin, a member of the serine protease inhibitor (serpin) superfamily, specifically inhibits human tissue kallikrein (Zhou *et al.*, 1992 ▸). Serpins are folded into a metastable conformation with a surface-exposed reactive-centre loop (Huber & Carrell, 1989 ▸; Gettins, 2002 ▸; Irving *et al.*, 2000 ▸). Once the reactive loop has been recognized and cleaved by the target protease, serpins undergo a dramatic conformational change in which the reactive loop is incorporated into the middle of the central β-sheet with translocation and inactivation of the covalently linked protease (Huntington *et al.*, 2000 ▸). This unique conformational change from a metastable to a hyperstable state, often termed a stressed-to-relaxed (S-to-R) conformational transition, is accompanied by a large free-energy change which is utilized to inhibit the protease (Huber & Carrell, 1989 ▸; Gettins, 2002 ▸).

Kallistatin is one of the heparin-binding serpins, which include antithrombin, protein C inhibitor, plasminogen activator inhibitor 1, heparin cofactor II and protease nexin I (Chen *et al.*, 2001 ▸; Gettins, 2002 ▸; Rein *et al.*, 2011 ▸; Patston *et al.*, 2004 ▸). The ability of heparin to enhance the inhibitory activity of serpins has been well studied (Gettins, 2002 ▸; Li *et al.*, 2004 ▸; Johnson & Huntington, 2003 ▸; Jin *et al.*, 1997 ▸; Dementiev *et al.*, 2004 ▸). For example, the binding of heparin to antithrombin increases its inhibition of thrombin or activated factor X by nearly 1000-fold. This is either through a template mechanism or an allosteric mechanism in which heparin binds to helix D of antithrombin (Li *et al.*, 2004 ▸; Jin *et al.*, 1997 ▸). Similarly, heparin can also accelerate the inhibition of activated protein C through binding to helix H of protein C inhibitor (Huntington & Li, 2009 ▸). Unexpectedly, it has been reported that heparin blocks the interaction between kallistatin and tissue kallikrein. The heparin-binding site has been proposed to be around helix H and strand 2 of β-sheet C of kallistatin (Chen *et al.*, 2001 ▸). As this unique heparin-mediated inhibition on kalli­statin is not well understood and the structure of kallistatin is not known, we have prepared and crystallized human kalli­statin as the first step towards elucidating the heparin-mediated regulatory mechanism.

## 2. Materials and methods   

### 2.1. Macromolecule production   

The kallistatin IMAGE cDNA clone was purchased from Geneservices and the coding sequence for amino acids 48–397 was amplified by PCR using gene-specific primers containing BamHI and HindIII sites (Table 1 ▸). The fragment was subsequently cloned into the pSumoO3 expression vector. The recombinant sequence was verified by DNA sequencing.

The construct contains a His-tagged Sumo3 at the N-terminus of kallistatin. The expression plasmid was transformed into *Escherichia coli* BL21(DE3) competent cells. A single colony was used to inoculate 60 ml LB medium, which was incubated overnight at 37°C with shaking. Cultured cells were then transferred to 6 l LB medium and incubated for 4 h at 37°C with shaking, after which the temperature of the culture was decreased to 20°C and protein expression was induced with 0.25 m*M* isopropyl β-d-1-thiogalactopyranoside for 20 h. Following induction, the bacteria were collected, resuspended and disrupted by sonication in 150 ml buffer *A* (20 m*M* Tris–HCl pH 7.5, 0.5 *M* NaCl, 20 m*M* imidazole). The bacterial cell lysate was subsequently centrifuged at 20 000*g* for 30 min at 4°C, after which the supernatant was applied onto a HisTrap Ni-Sepharose column (5 ml). The unbound bacterial proteins were washed from the column using 100 ml buffer *A*. The target protein was subsequently eluted from the column using a 20–200 m*M* imidazole gradient in buffer *A*. Fractions containing the fusion protein, based upon SDS–PAGE analysis, were pooled and then digested with SENP2 protease to remove the Sumo3 tag from the fusion protein and to generate recombinant kallistatin without the non-native sequence at its N-terminus. The mixture was dialyzed and loaded onto a HiTrap SP ion-exchange column. Kallistatin was eluted with a 0–1 *M* NaCl gradient in 10 m*M* MES pH 6.2. Peaks containing kallistatin were collected and concentrated before crystallization trials.

### 2.2. Crystallization   

The initial conditions for crystallization were screened at 22°C by the sitting-drop vapour-diffusion method using screening kits from Hampton Research (Crystal Screen, Crystal Screen 2, Index HT, PEG/Ion, PEG/Ion 2, SaltRX, Natrix, MembFac and Crystal Screen Cryo) in MRC2 plates. Crystals were initially grown from a mixture of 200 nl protein solution (10 mg ml^−1^ in 10 m*M* MES pH 6.2, 0.15 *M* NaCl) and 200 nl precipitant solution equilibrated against 80 µl reservoir solution. Subsequent optimizations were performed using 24-well sitting-drop plates and the crystals grew to dimensions of 0.2 × 0.2 × 0.5 mm in two weeks. A summary of the crystallization is provided in Table 2 ▸.

### 2.3. Data collection and processing   

Owing to the rapid evaporation of *tert*-butanol, the crystals float around in the drop and crack once the well is opened. A cryosolution consisting of 10% glycerol and 10% ethylene glycol was directly added to the drop and a small piece of crystal was picked up using a micro-loop and flash-cooled in liquid nitrogen. Diffraction data were collected on beamline BL17U at SSRF, Shanghai, People’s Republic of China. The data set was indexed and processed with *iMosflm* (Battye *et al.*, 2011 ▸) and scaled with *AIMLESS* from the *CCP*4 suite (Evans, 2011 ▸; Winn *et al.*, 2011 ▸).

## 3. Results and discussion   

**Figure 1:**
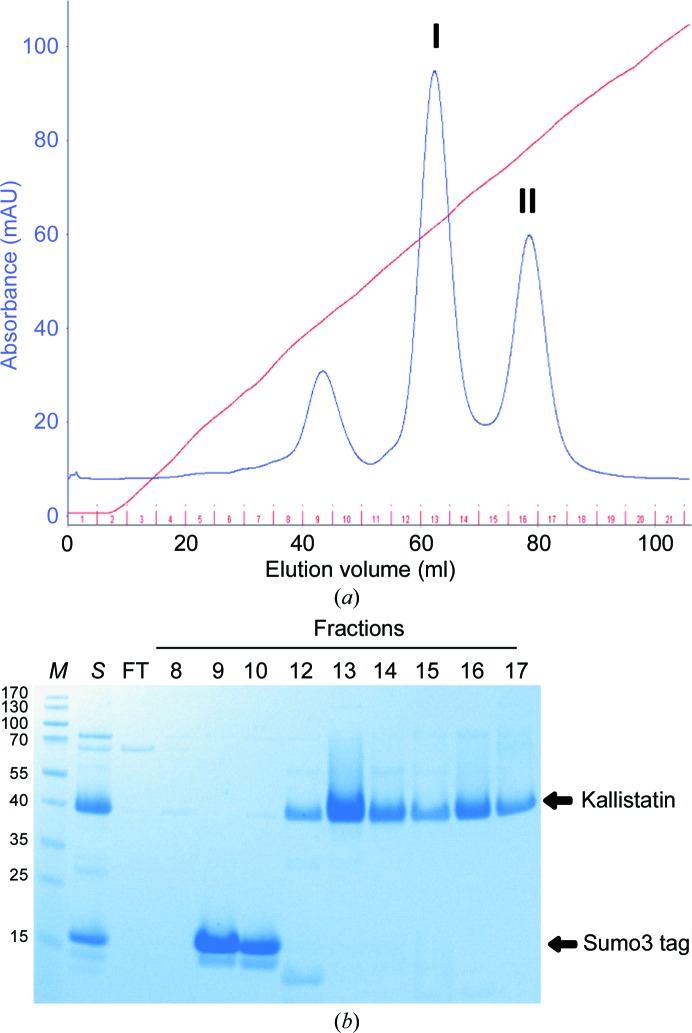
Purification of recombinant kallistatin by ion-exchange chromatography. The Sumo3-kallistatin fusion protein was cleaved with SENP2 and the mixture (lane *S*) was loaded onto a HiTrap SP column. The protein was eluted with an NaCl gradient from 0 to 1 *M*, measuring the absorbance at a wavelength of 280 nm (*a*). Flowthrough (FT) and fractions from elution were analysed by SDS–PAGE (*b*). Fractions from peak I (13 and 14) and peak II (15–17) were pooled separately and subjected to crystallization trials. Lane *M* contains molecular-weight marker (labelled in kDa).

**Figure 2:**
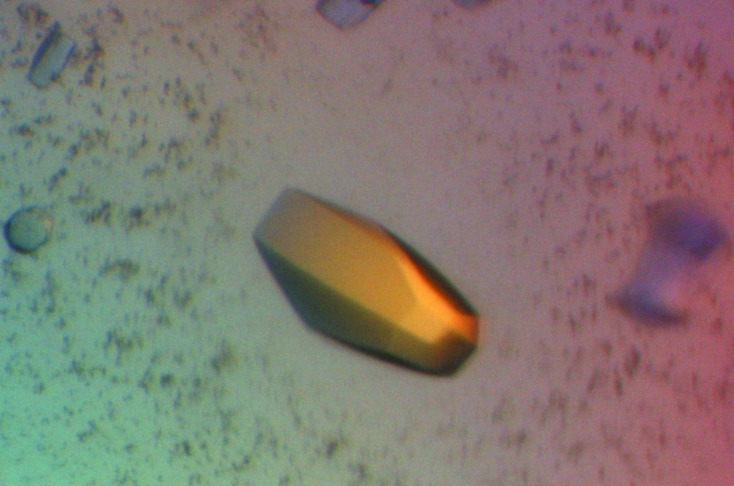
Crystals of human kallistatin grown in 30% *tert*-butanol belonged to space group *P*6_1_, with unit-cell parameters *a* = 113.51, *b* = 113.51, *c* = 76.17 Å.

In order to understand the molecular mechanism of kallistatin in regulating protease activity, we expressed recombinant human kallistatin as a fusion protein using an *E. coli* expression system. After Ni-Sepharose affinity-column purification, the fusion tag was cleaved with SENP2 protease and kallistatin was further purified on a cation-exchange column. As shown in Fig. 1 ▸, the Sumo3 tag eluted in fractions 9 and 10 and kallistatin eluted as two peaks (I and II). Proteins from both peaks were pooled and screened for crystallization. Stick-shaped crystals were obtained with protein from peak II after two weeks using 30% *tert*-butanol as precipitant (Fig. 2 ▸). These crystals readily diffracted to better than 2 Å resolution and were found to belong to space group *P*6_1_, with unit-cell parameters *a* = 113.51, *b* = 113.51, *c* = 76.17 Å. Diffraction data statistics are shown in Table 3 ▸.

**Figure 3:**
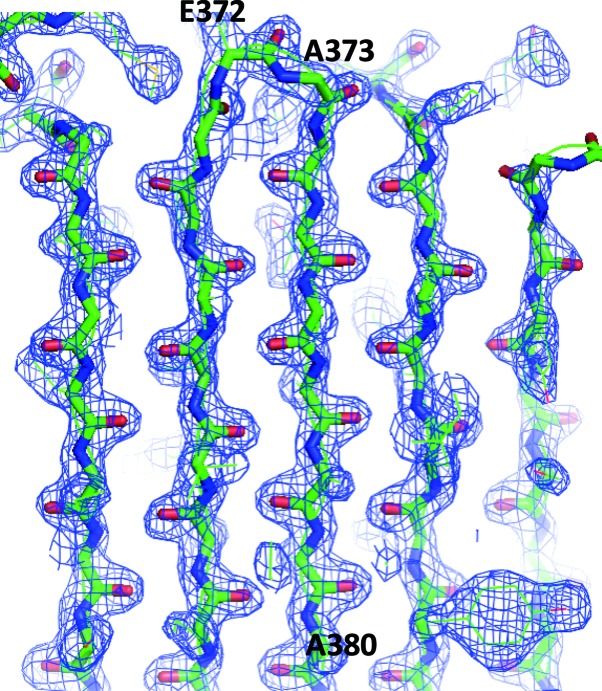
Electron-density map from *Phaser* showing that the reactive loop of kallistatin (residues 373–380) is inserted into the central β-sheet. The map is contoured at 2σ and the image was prepared using *PyMOL* (http://www.pymol.org).

Molecular replacement was performed with *Phaser* (McCoy *et al.*, 2007 ▸). As serpins are known to adopt different conformations, models of protein C inhibitor (Huntington *et al.*, 2003 ▸; Li *et al.*, 2007 ▸) and thyroxine-binding globulin (Zhou *et al.*, 2006 ▸), representing one relaxed and two stressed serpin conformations (∼47% sequence identity; PDB entries 1lq8, 2ce0 and 2hi9), were selected as search models. A clear single solution was obtained from a search using PDB entry 1lq8 as the model with one copy of molecule in the asymmetric unit, indicating a solvent content of ∼60%. As PDB entry 1lq8 corresponds to the relaxed conformation of protein C inhibitor with its reactive loop cleaved and inserted into the central β-sheet (Huntington *et al.*, 2003 ▸), this indicates that the crystallized kallistatin is also in a relaxed conformation. The electron-density map calculated from *Phaser* confirms that the reactive loop of kallistatin is incorporated into the central β-sheet A as a middle strand (Fig. 3 ▸). Therefore, kallistatin is likely to have undergone conformational changes to form the relaxed conformation during expression or during the sub­sequent purification and crystallization steps. Preliminary refinement with *REFMAC* (Murshudov *et al.*, 2011 ▸) using the model from *Phaser* gave an *R*_work_ of 33% and an *R*_free_ of 37%. Further refinement and detailed characterization of the cleavage site are ongoing.

## Figures and Tables

**Table 1:** Macromolecule-production information The restriction-enzyme cleavage sites are underlined.

Source organism	*Homo sapiens*
DNA source	GenBank L19684.1
Forward primer	5-GTTTGGATCCAGCCTCAAGATAGCCCCTG-3
Reverse primer	5-CAAAAAAGCTTATGGTTTCGTGGGGTCGAC-3
Cloning vector	pSumo3
Expression vector	pSumo3
Expression host	*E. coli* BL21(DE3)
Complete amino-acid sequence of the construct produced	SLKIAPANADFAFRFYYLIASETPGKNIFFSPLSISAAYAMLSLGACSHSRSQILEGLGFNLTELSESDVHRGFQHLLHTLNLPGHGLETRVGSALFLSHNLKFLAKFLNDTMAVYEAKLFHTNFYDTVGTIQLINDHVKKETRGKIVDLVSELKKDVLMVLVNYIYFKALWEKPFISSRTTPKDFYVDENTTVRVPMMLQDQEHHWYLHDRYLPCSVLRMDYKGDATVFFILPNQGKMREIEEVLTPEMLMRWNNLLRKRNFYKKLELHLPKFSISGSYVLDQILPRLGFTDLFSKWADLSGITKQQKLEASKSFHKATLDVDEAGTEAAAATSFAIKFFSAQTNRHILRFNRPFLVVIFSTSTQSVLFLGKVVDPTK

**Table 2:** Crystallization

Method	Sitting-drop vapour diffusion
Plate type	MRC2 plates, 96-well
Temperature (K)	295
Protein concentration (mgml^1^)	10
Buffer composition of protein solution	10m*M* MES pH 6.2, 0.15*M* NaCl
Composition of reservoir solution	35% *tert*-butanol
Volume and ratio of drop	0.4l; 0.2:0.2
Volume of reservoir (l)	80

**Table 3:** Data collection and processing Values in parentheses are for the outer shell.

Diffraction source	BL17U, SSRF
Wavelength ()	1.196
Temperature (K)	100
Detector	ADSC Q315
Crystal-to-detector distance (mm)	250
Rotation range per image ()	1
Total rotation range ()	60
Exposure time per image (s)	0.5
Space group	*P*6_1_
*a*, *b*, *c* ()	113.51, 113.51, 76.17
, , ()	90.00, 90.00, 120.00
Mosaicity ()	0.45
Resolution range ()	35.71.9 (2.01.9)
Total No. of reflections	185915 (26532)
No. of unique reflections	44414 (6433)
Completeness (%)	99.9 (99.7)
Multiplicity	4.2 (4.1)
*I*/(*I*)	14 (2.5)
*R*_merge_	0.043 (0.326)
*R*_meas_	0.049 (0.374)
Overall *B* factor from Wilson plot (^2^)	30.1
